# Dr. F. D. Wilson

**Published:** 1899-02

**Authors:** 


### OBITUARY.

### Dr. F. D. Wilson.

Died in Findlay, Texas, January 1, 1899, Dr. F. D. Wilson of hæmorrhage of the lungs. Dr. Wilson was a graduate of the Dental Department of the University of Michigan in 1881. He had been practicing in Omaha, Neb., for some time. The Doctor had been in feeble health for a year or two, was on his way to Mexico for his health, but he succumbed to the disease before he arrived at his destination.

Dr. Wilson was a model man. All who knew him entertained the highest regard for him as a man and as a dentist.

